# Participatory action research to co-design a culturally appropriate COVID-19 risk communication and community engagement strategy in rural Pakistan

**DOI:** 10.3389/fpubh.2023.1160964

**Published:** 2023-04-24

**Authors:** Victoria Hall Moran, Marena Ceballos-Rasgado, Sadia Fatima, Usman Mahboob, Salman Ahmad, Michael McKeown, Mukhtiar Zaman

**Affiliations:** ^1^Centre for Global Development, University of Central Lancashire, Preston, United Kingdom; ^2^Institute of Basic Medical Sciences, Khyber Medical University, Peshawar, Pakistan; ^3^Institute of Health Professions Education and Research, Khyber Medical University, Peshawar, Pakistan; ^4^Department of Sociology, Abdul Wali Khan University, Mardan, Pakistan; ^5^School of Nursing, University of Central Lancashire, Preston, United Kingdom; ^6^Department of Pulmonology, Rehman Medical Institute, Peshawar, Pakistan

**Keywords:** participatory action research, COVID-19, risk communication and community engagement, health promotion, infectious diseases, global health

## Abstract

**Background:**

Community engagement has shown to be fundamental component of the response to previous disease outbreaks. This study aimed co-design and implement a culturally appropriate COVID-19 risk communication and community engagement strategy with a resource-poor rural community in Northwest Pakistan.

**Methods:**

Participatory Action Research (PAR) was conducted from January 2021 to March 2022. Five PAR meetings took place with community members (*n* = 30) to: (1) explore how the COVID-19 pandemic impacted on the community; (2) identify challenges to limit the spread of the virus; (3) identify and implement solutions to these challenges; and (4) highlight the enablers, challenges and knowledge of the cultural context needed to optimize safety during emergencies. Focus group discussions (*N* = 6) with community members not involved in the PAR meetings (*N* = 50) and children of the community (*N* = 26) were conducted following the PAR meetings. Thematic analysis of the PAR and focus group data was conducted.

**Results:**

Delivery of messages on how to keep families safe, provision of personal protective equipment and improved water systems were part of the strategies taken by the community to create awareness and reduce the spread of COVID-19. Nine themes were identified: Attitudes to the pandemic: From skepticism to acceptance, Changing attitudes about vaccination: rumors and trust, COVID-19 and Faith, Social impact of the pandemic, Access to water, Resource mobilization: personal protective equipment, Spaces where collaborative effort can bring to solutions, Agents of change, and Empowerment of women.

**Discussion:**

The participatory approach of this research allowed understanding of the challenges faced by the community to engage in behavior change strategies to reduce the spread of COVID-19 and enabled the community to find sustainable solutions. Engagement with the community empowered men and women to be agents of change and promoted necessary precautionary actions to reduce the risk of infection within their community.

**Conclusion:**

Participatory approach highlighted the importance of engaging with and integrating to local culture and values to overcome challenges such as gender imbalance and distrust. Findings of this study are relevant to others working in diverse cultural settings in similar crises events regardless of particular cultural variations.

## Introduction

In January 2020, the World Health Organization (WHO) declared the novel coronavirus outbreak (SARS-CoV-2) a public health emergency of international concern and in March 2020 pronounced this a pandemic (1). Globally, there have since been more than 600 million cases of COVID-19 and over 6.5 million deaths (2). COVID-19 has led to unprecedented international public health measures, and as governments around the world declared states of emergency, issued stay-at-home orders, and advised avoidance of social gatherings, hundreds of millions of individuals found themselves out of work, in both formal and informal labor markets (3). Low-and middle-income countries’ (LMIC) capacity to respond to such health emergencies is typically constrained, as they face a double burden of disease and resource limitations. With a public health system already under severe strain, and an acute shortage of doctors and nurses and an absence of universal health coverage, the pandemic exacerbated the health, economic and social vulnerabilities of Pakistan’s population (4). Adherence to COVID-19 public health recommendations was particularly challenging for those living in rural areas with low daily incomes and limited access to clean water, sanitation, and hygiene facilities (WASH). Effective and meaningful risk communication strategies during this period of rapidly evolving information were vital to minimize the spread of the virus (5).

It has long been recognized that the complexities and challenges of supporting public health in developing nations demands that services and workforce are increasingly responsive to the realities of local context, crucially including the expressed needs of communities (6). Sustainable and locally owned solutions and practices are therefore essential to addressing public health issues. Community engagement has shown to be an effective strategy to developing the potential of communities (7) that has a positive impact on health outcomes, improves knowledge of health issues and participation in health screening programs (8–11). Following the Ebola outbreak in 2014, the United Nations published a Global Response to Health Crises recommending strengthening community engagement to promote local ownership and trust in future crisis response (12). In March 2020, the World Health Organization (WHO) published their Risk Communication and Community Engagement (RCCE) Action Plan Guidance to Support COVID-19 Preparedness and Response. The purpose of this guidance was to support risk communication, community engagement staff and other partners in developing, implementing, and monitoring an action plan that would allow effective communication and engagement with the public and local partners to help prepare and protect individuals, families, and the public’s health during early responses to COVID-19 (13). A key consideration to the guidance was adapting the action plan to specific country needs and situations.

The aim of this participatory action research study was to co-design and implement a culturally appropriate COVID-19 risk communication and engagement strategy with a rural community in Northwest Pakistan. Specific objectives were to: (i) explore how the COVID-19 pandemic impacted on the lives of the community; (ii) identify challenges to following public health recommendations to reduce the spread of COVID-19 within the community; (iii) identify and implement a strategy to bring solutions to some of the challenges faced by the community to keep their family safe during the pandemic; and iv) provide guidance on the enablers, challenges and cultural context to optimize safety during public health emergencies.

## Methods

### Study design

**Figure 1:**
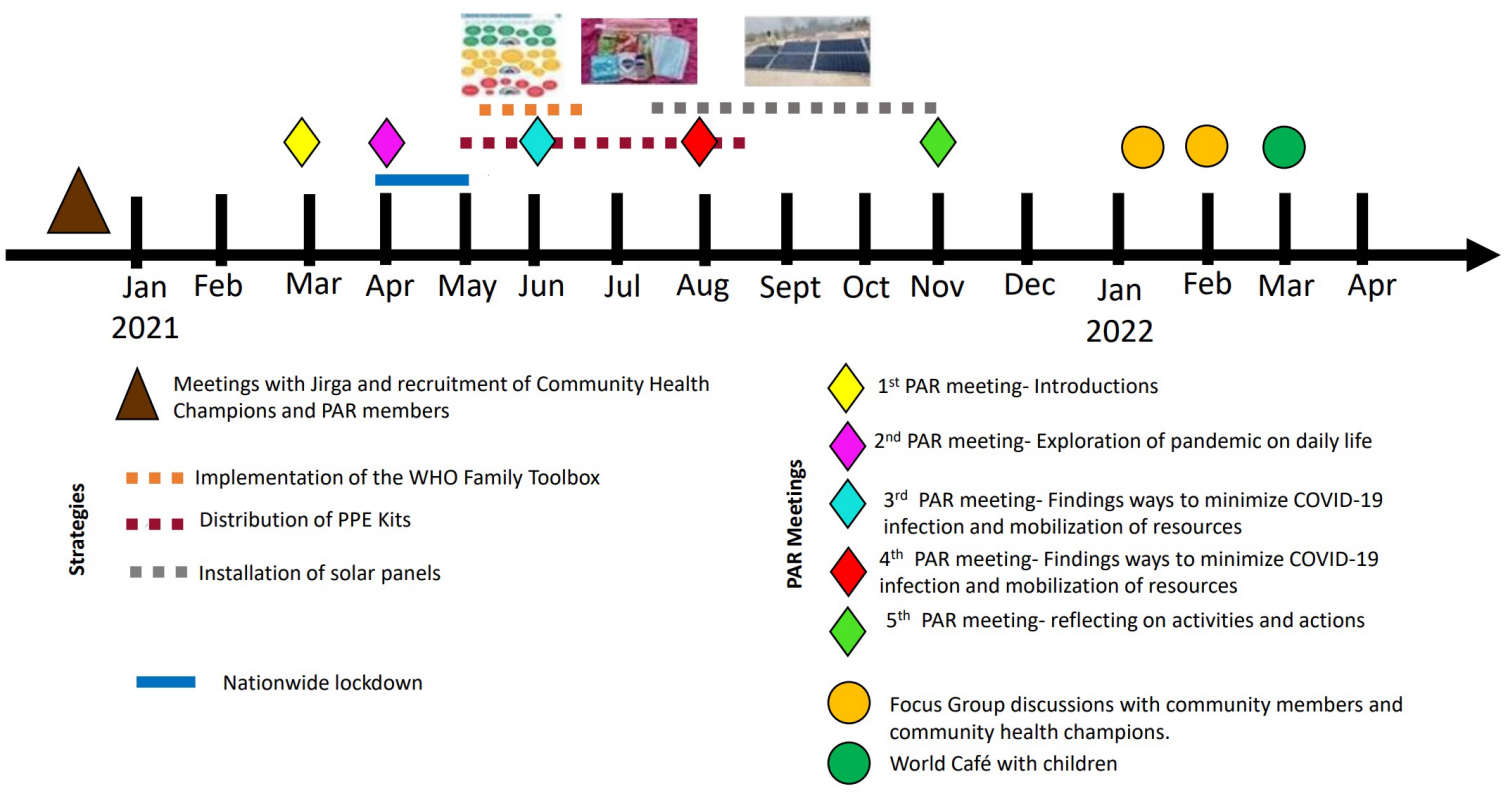
Timeline showing the different phases of the project.

This study adopted a qualitative community-based participatory action research (PAR) approach (8) to meet the objectives listed above. Participatory action research is a flexible approach to research that involves researchers and community members working together to identify relevant social problems or issues in need of change in their communities. The intention is to formulate and implement practical solutions leading to improvements in social conditions. The PAR approach proceeds in iterative, deliberative cycles of planning, action, and reflection (see Figure 1). The ethos is essentially democratic and dependent upon the quality of communication transacted between participants; done well, the approach supports empowerment of disadvantaged communities, boosting the confidence and agency of participants toward achieving agreed goals (14, 15). In this project, the participants were empowered to make decisions regarding mobilization of tangible resources to enhance their sense of agency and action. To support this the community had access to a fixed amount of funding (£40,000) with which they were able to prioritize and mobilize material resources to enable them to meaningfully operationalize key public health practices. The study comprised four core elements: (1) PAR meetings with members of the community to co-develop a strategy to reduce the spread of COVID-19 within their community; (2) Resource mobilization based on needs identified by the PAR members; (3) Delivery of safety messages through Community Health Champions (CHCs) and (4) Focus group discussions (FGDs) with the wider community to evaluate the impact of the initiative.

### Materials

**Figure 2:**
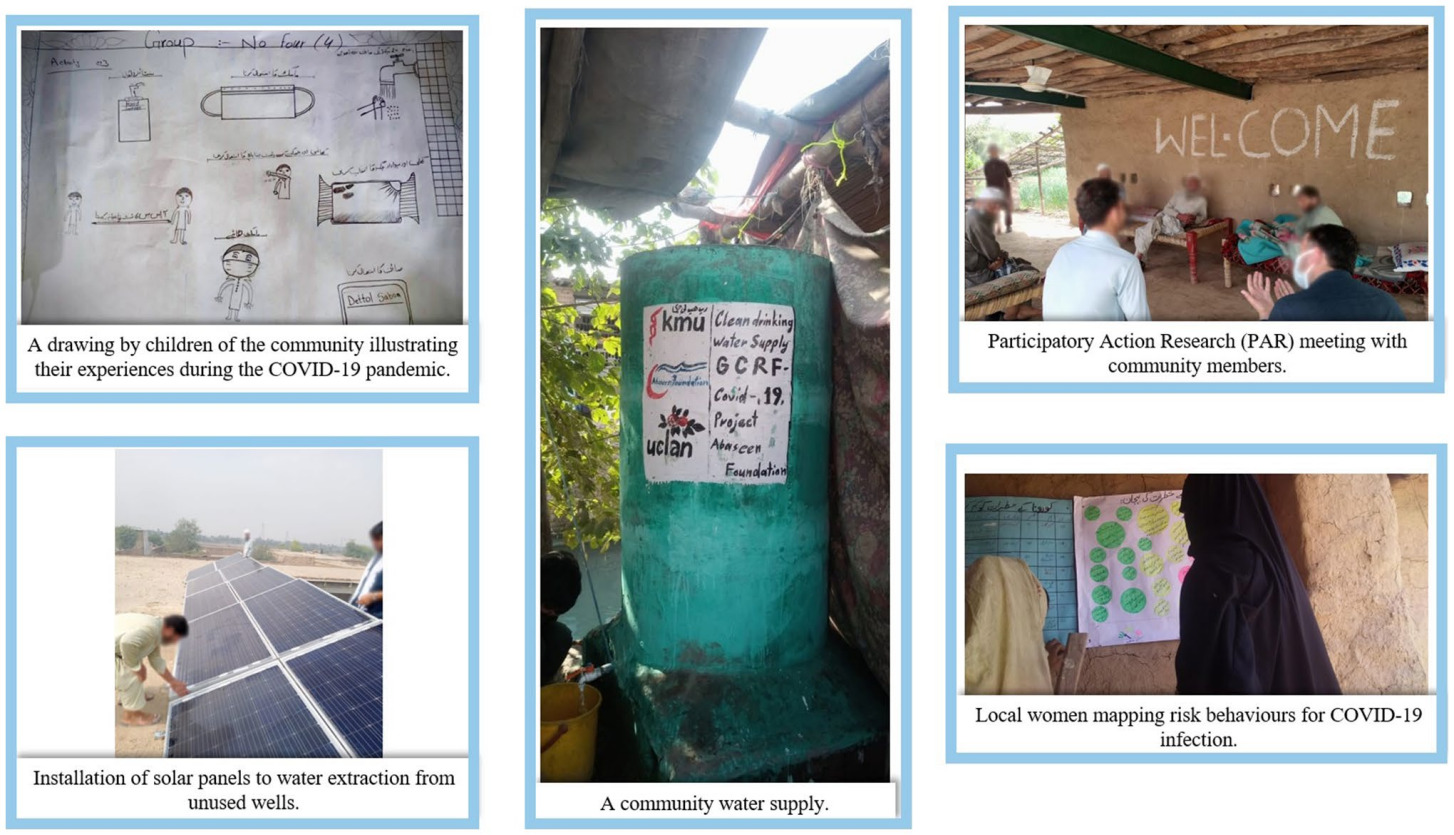
Photographs illustrating the different elements of the project.

We used the WHO Family Toolbox for Managing Health and Happiness During COVID-19 (16) as a resource to facilitate the delivery of public health messages. The WHO Family Toolbox aims to bring families and households together to manage shared risks and agree to safe behaviors critical for their safety and the safety of their community. It places family at the center of all activities, in recognition that the family is a critical influencing structure that can promote decisions with potential to shift norms within the family and ultimately their behaviors. CHCs worked with families to map their daily activities and interactions in their community to help them identify which activities they could modify, avoid or reduce to minimize their exposure to the virus (see Figure 2). Following discussions with the Department of Health RCCE lead in Khyber Pakhtunkhwa we modified the toolkit to include information pertaining to vaccination.

### Setting

The setting for this research was a remote, rural community consisting of around 500 households situated 25 km southeast of the city of Peshawar, Khyber Pakhtunkhwa (KP) Province, Northwest Pakistan. This region is one of the most disadvantaged areas of a disadvantaged nation, with our study centered upon the brick-kiln settlements known for high levels of poverty, illiteracy, indentured labor, internally displaced people, and associated health deficits. The community has limited access to clean water, sanitation, and healthcare. Gender inequality is of significant concern in Pakistan, and in this traditionally tribal and patriarchal culture, women’s movements outside of the home are severely restricted. Our project was centered around a school in the area, one of the three schools for boys and girls that was set up and run by the Abaseen Foundation. In an area where adult female literacy is less than 3%, these schools represent a shift in cultural attitudes to female education in this community. This locus had the potential to soften the patriarchal culture of the area and provided an opportunity to recruit both mothers and fathers of children who attended the school.

### Participants and procedures

#### PAR groups

We formed two PAR groups (one male and one female), recruited from families whose children attended the local school with a total of 15 participants in each group. Our intention was to recruit participants who were active and vocal members of the community, and this was supported by advice from the NGO working in the community with the help of Jirga members using snowball sampling. To be eligible participants had to be 18 years old or older, be willing to participate in at least four of the five planned meetings, and have the ability to provide informed consent. Male and female participants were belonged to the same family groups, for example, 12 of the 30 PAR group members were spousal partners, overcoming any potential cultural barriers to participation.

A total of ten PAR meetings (five with men and five with women) (see Figure 2) were conducted between March 2021 and November 2021. A structure for each meeting was planned *a priori* but this was kept flexible to allow PAR members to lead on agenda and discussions. These were structured as follows: PAR meeting 1, introductions, ice breaking and setting mutually agreed ground rules around respect, trust and confidentiality of information shared in the meetings; PAR meeting 2, exploration of knowledge, attitudes and perceptions about COVID-19 and the impact of the pandemic on daily life; PAR meetings 3 and 4, exploration of perceived risks and challenges to prevent COVID-19 infection within households, and decisions about the mobilization of resources; and PAR meeting 5, reflection on the activities and actions of the group and discuss further work and sustainability. Research assistants, who received training from researchers at UCLan and KMU on PAR methodology, facilitated the PAR meetings in the local language. PAR meetings took place in open spaces within the communities to reduce risk of infection of COVID-19 and PAR members were advised not to attend the meetings if they had any symptoms of COVID-19. Each PAR meeting was designed to last approximately 60 min.

### Community health champions

The participatory processes, community engagement and dissemination activities were supported by Community Health Champions (CHCs) who had a key role in linking the PAR members with the community, operating as a two-way vector for information exchange. Five (three men and two women) CHCs were recruited on a paid role through the NGO between March 2021 and November 2021. CHCs resided in the local area, were Pashto speakers and literate, aged between 15 and 50 years, and had knowledge of the challenges faced by the local community. Using the WHO Family Toolbox for Managing Health and Happiness during COVID-19 (16), five CHCs delivered information about how to limit the spread of COVD-19 and explained how they could use the Toolbox to identify the activities that the families may encounter during their day which presented a risk for contracting COVID-19 and develop a plan to reduce this risk. The CHCs were invited to take part in a FGD 3 months following the end of their contract to explore their experiences of their role in this study.

#### Community members

Four focus group discussions, two with men (*N* = 25) and two with women (*N* = 20), were held following the conclusion of the PAR group meetings and associated resource mobilization activities. The purpose of the FGDs were to explore the community members’: (i) awareness of the project; (ii) views about whether the PAR groups and CHCs adequately identified community needs; and (iii) views and experiences of the associated resource mobilization activities. Research assistants recruited the community members through snowball sampling. To be eligible participants were 18 years old or older and be able to provide informed consent.

Children who attended the local school were also invited to share their experiences of the pandemic and of the project, adopting a World Café approach. This approach makes use of an informal setting where participants explore an issue by discussing it in small groups. Research assistants met with the schoolteachers, parents, and children in Grade 5 (10 to 11 years) and the aim of the study was explained. Oral and written consent was obtained from parents. Discussions with the children, using separate groups for boys and girls, were held in four rounds of 20 min.

The FGDs and World Café were led by trained research assistants. Topic guides for the FGDs and the World Café were collaboratively developed by researchers at UCLan and KMU and translated into the local language. Topic guides are presented in Supplementary material 1.

### Public involvement

Our chosen methodology, participatory action research, shares a common philosophy with PPI of valuing partnership and collaboration. Co-creation of the research question and dissemination of findings is embedded within the long-term relationship between the project partners and achieved through ongoing engagement with the community and Jirga (respected male elders) and in accordance with the Global Code of Conduct for Research in Resource-Poor Settings (17). The community informed the study from the outset, communicating the need for the study through our existing collaborative networks. Using PAR methods, we engaged community members and community leaders in the design and conduct of the study and it was the community who decided on the study outcomes that were important to them. Recruitment was conducted with the assistance of the Jirga and community health champions disseminated ongoing study findings and discussion points to the wider community throughout the project.

### Data analysis

The PAR meetings, FGDs and World Café were audio recorded and translated and transcribed into English by transcribers who have excellent understanding of both the local language and English. A senior researcher at KMU (UM) double-checked the transcripts for accuracy. All participant names or identifying information were removed to maintain participant anonymity. Qualitative data was coded and thematically analyzed concurrently by researchers in Pakistan and the UK using qualitative data analysis software NVivo version 11. For data analysis, we followed the six-step to thematic analysis proposed by Braun and Clarke following a semantic and inductive, coding and theme development (18).

Two researchers (one UK based, MCR and one Pakistan based, UM) independently familiarized themselves with the data, generated initial codes, searched for themes, and defined and named initial themes. Candidate themes and interpretations were presented to the wider team and compared. Any differences were resolved through discussion and feedback. Upon feedback from all authors, themes were refined, and transcripts re-read and re-coded. The data extracts that best reflected the themes were chosen as illustrative examples in this article. The data files were shared, and two researchers VHM & McK provided suggestions and feedback to further remodify them. The first and second authors shared and presented the final data to all authors for their agreement.

To write this manuscript, the consolidated criteria for reporting qualitative research (COREQ) (19) and the Sex and Gender Equity in Research (SAGER) guidelines (20), were used to ensure accurate and complete reporting of the study context, methods, data analysis, findings, and their interpretations.

### Ethical considerations

Literate participants were given a Participant Information Sheet, a consent form and COVID-19 Research Participant Pre-Visit Check form to identify individuals who may be at greater risk of transmitting the virus during the recruitment process. Participants were asked not to attend the group (PAR, FGD, World Café) if they had recently been exposed to someone with COVID-19 or had symptoms indicative of COVID-19. The documents were written in Urdu and were read those participants who were not literate, who then indicated their consent by signing with their initials or an X. The study participants were allotted identity numbers during the selection process to keep their identity anonymized. Participants were informed of their rights to participate and that they could withdraw from the study at any time. All participants were given a gift (cost: USD 15) as a reward for their participation on the study.

## Results

Following conclusion of the PAR groups and associated mobilization of resources, six FGDs were held: one with CHCs (*n* = 3 men and *n* = 2 women), two with male community members (*n* = 25), two with female community members (*n* = 20), and a one (conducted as a World Café) with children (3 male groups, *n* = 12, and 3 female groups, *n* = 14) to elicit their perspectives of the PAR process and their experiences during the pandemic. Sociodemographic characteristics of the participants are presented in Table 1.

**Table 1:** Sociodemographic characteristics of the participants.

Factor	PAR group	Focus group	World Café (Children)	CHCs
*N*	29^1^	45	26	5
Age (SD)	42.6 (9.26)	37.55 (10.60)	–	–
Sex Female (%)	15 (50)	20 (44.4)	14 (53.85)	2 (50)
Marital status (%)				
Married	28 (96.55)	44 (97.78)	–	–
Unmarried	1 (3.44)	1 (2.22)	–	–
Monthly Income (%)^2^				
< 70.00 USD	12 (52.17)	33 (73.33)	–	–
70–141 USD	4 (17.39)	7 (15.56)	–	–
> 141 USD and above	7 (30.43)	5 (11.11)	–	–
*Source of drinking water (%)* ^2^ ^,^ ^3^				
Well	11 (50%)	16 (35.56)	–	–
Tube Well	7 (31.82)	21 (46.67)	–	–
Other	4 (18.18%)	8 (17.78)	–	–

1 One participant deceased (non-covid reason).

2 Twelve PAR group participants were married couples and only data from one household is presented.

3 Data missing from one PAR group participant.

We enquired about the challenges faced during the COVID-19 pandemic and the community-decided implemented actions that were carried out to reduce its transmission. For the purpose of this paper, a thematic analysis of the PAR meetings and focus group discussions with community members and community health champions are presented conjointly to find commonalities and differences across participants’ perspectives. Effectively, the findings of a PAR project are an amalgam of the actions resulting from PAR deliberations and analysis of the process by which these were achieved.

**Figure 3:**
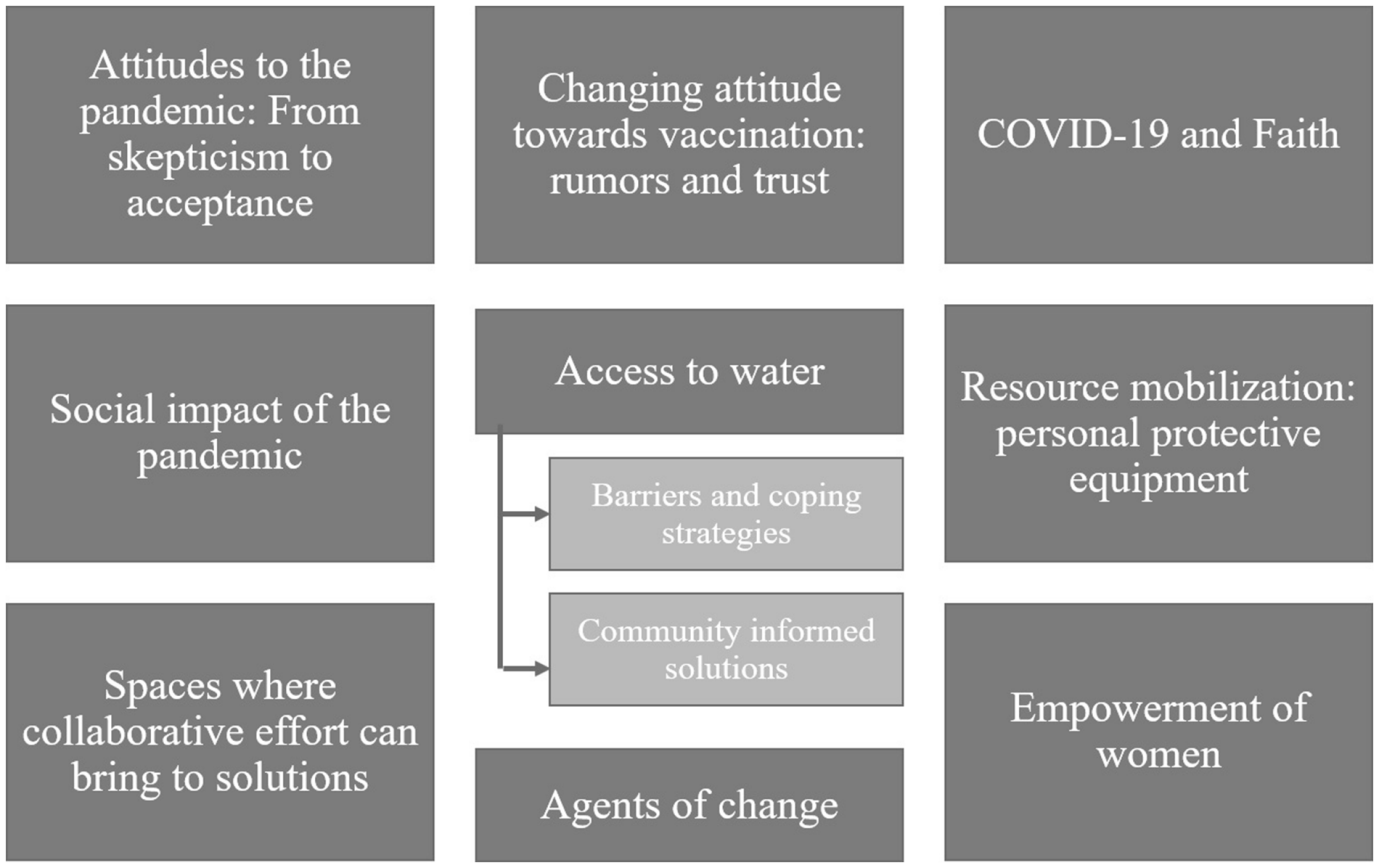
Key themes from the thematic analysis of the PAR meetings, focus group discussions and world café method with community members and health champions.

A total of nine key themes were identified as shown in Figure 3.

### Attitudes to the pandemic: from skepticism to acceptance

Across all the groups (PAR meetings, focus groups with community members, CHCs and children) participants reported that there were some people within their community that disputed the existence of the pandemic. Some of them expressed that people believed that the COVID-19 pandemic was a contrivance originating from the West to keep people apart and harm their culture and religion. These attitudes were also held by some of the PAR participants:

*“We have been distant from each other forcefully. There is no corona [COVID-19]. They are trying to put a stop to the weddings and charities we give, but instead, we want harmony among each other.” Woman, 2nd PAR meeting*

The differing views in the community toward whether or not the pandemic existed caused confusion among some participants. Despite this, they felt the need to protect themselves and their families although they expressed uncertainty regarding what action to take:

*“We don’t know what is going to happen, we don’t even know whether corona [COVID-19] exists or not. We don’t know what is good or bad for us. We pray and go to weddings, funerals and mosques this is very hard for all of the Muslims to follow each in every SOP [standard operating procedure] of the government.” Man, 2nd PAR meeting*

The attitudes toward the existence of COVID-19 and attendant risk changed throughout the course of this project and this was reflected in our data. One of the factors that contributed toward this realization was experiencing the disease themselves or witnessing people from their community falling ill or dying:

*“My brother bought masks for us while the villagers thought it was a conspiracy against Muslims. But when they saw the people dying due to it, they believed it was a real pandemic.” Girl, World Cafe*

Those engaged directly in the project believed they were pivotal in helping to change attitudes in the community. For example, a CHC felt that their participation in the project helped to raise awareness among members of the community about the importance of taking precautionary measures:

*“We soon realized that it is a pandemic and is very harmful, so we decided to save ourselves and our families from it … PAR meetings were organized which gathered different people from the societies … We met them at shops and gatherings.. we asked them to wear masks and sanitize their hands. After a few meetings people were convinced that COVID-19 pandemic exists.” CHC, FGD*

### Changing attitude toward vaccination: rumors and trust

There were differing opinions within the community regarding vaccination. Participants expressed that there was an initial vaccine hesitancy, and some were influenced by hearing rumors about its safety:

“Some say people will die within one year. Some people say it protects us from the corona *[COVID-19]* … Some people say that people can’t conceive children.” Woman, 3rd PAR meeting

During the later PAR group meetings, participants believed that there was change of attitudes toward vaccination within their community and alleged this helped to reduce the number of cases of COVID-19:

*“The death ratio of the corona [COVID-19] is less in our area than in other regions due to the efforts of (NGO name). Many people got vaccinated in this area.” Man, 4th PAR meeting*

Some PAR members and CHCs expressed that their intention to get vaccinated changed due to the information received during the project and due to seeing other respected people getting vaccinated without harm:

“Before we were not ready to be vaccinated. We saw our seniors and managers getting vaccinated and when we saw them perfect, we also got vaccinated.” CHC, FGD

### COVID-19 and faith

During the first and second PAR meetings some of the participants drew upon spiritual understandings grounded in their Islamic faith:

*“Corona [COVID-19] is upon us because of our sins. Not even a single leaf moves without the will of Allah. And in this we have faith … In a family, if a person falls sick everyone in the family helps him to recover as being a Muslim or a human and thanks to Allah that no one else got infected from it.” Man, 2nd PAR meeting*

For some participants their faith underpinned a commitment to protect themselves by following precautionary measures in accordance with religious norms of cleanliness, or that ablution would protect them against the disease:

*“We can protect ourselves from the coronavirus by praying 5 times a day, and ablution. Wash our hands at regular intervals. It is very warm these days and we should take baths more often, use sanitizer and wearing masks.” Woman, 3rd PAR meeting*

### Social impact of the pandemic

PAR and focus groups participants expressed how COVID-19 impacted upon their lives. Some of the men talked about how COVID-19 affected businesses, whereas children’s concerns centered on their inability to meet with their family and friends.

*“Means the business have been affected a lot. In the Holy month of Ramadan, the shops were closed.” Man, 3rd PAR meeting*

*“The difficult thing was that we could not attend weddings or go to our family member’s houses. Neither could we play outside.” Girl, World café*

Overall, women were more vocal on how COVID-19 impacted their lives and some described how the lockdown represented a threat to their family welfare, impacting economically leading to unemployment and price increases:

*“A product worth Rs.5 is now being sold at Rs. 10 and Rs. 12 these days. It is the month of fasting [Ramadan] and our men are still jobless.” Woman, 2nd PAR meeting*

Women were also concerned that education should not be stopped for their children:

*“Our Madrasas [educational institutions] have been closed. All the children are at the home. They cannot go to schools and colleges as they are closed too. This is a waste of time for the children.” Woman, 2nd PAR meeting*

For other women, issues such as reducing crime, securing the household economy, children continuing their education, and low access to water, sanitation and power, and domestic violence took precedence over the fear of COVID-19 infection during lockdown:

*“The government says that corona exists, and we oppose it. In the mobiles, we see videos of boys who have been killed or thrown acid etc. they should take care of that first.” Woman, 2nd PAR meeting*

### Access to water

The theme access to water was constituted by two subthemes: (1) Barriers and coping strategies and (2) Community and informed solutions.

### Access to water: barriers and coping strategies

Participants described the barriers they faced to enable them to follow the public health guidance to prevent COVID-19. Water scarcity was a serious issue in the community, limiting their ability to stay clean, and, combined with other environmental issues, resulted in widespread health problems for families:

*“The scarcity of water is haunting us. Due to poverty, we are unable to buy soaps or have water. If we have water, we can clean ourselves and stop different diseases and flies.” Woman, 3rd PAR meeting*

Participants, including adults and children, described some of the coping strategies they would use to deal with the scarcity of water. They reported washing themselves less regularly, traveling further distances to obtain water, using old water, and obtaining water from the canal, recognizing that some of this heightened their risk of infection:

*“The people of this area could not find water even to wash their hands after using toilets, and they used to wash their hands with the water of the canal.” Man, FGDs*

*“We also had little water, which we would bring from far off areas. This was another reason for getting exposed to it [Covid-19].” Girl, World Cafe*

### Access to water: community informed solutions

During the course of PAR deliberations, the participants discussed possible solutions to improve access to water. This included installation of pipes, water pumps, and bores. Yet, some of the participants highlighted that the problem of water scarcity was also linked to a lack of electricity powering the water pumps to extract water from the wells. Installation of solar panels on unused wells was proposed as a possible solution to the water scarcity problem (see Figure 2):

*“The scarcity of water is a big issue of debate, but the biggest problem is that of electricity. The solution to our problems is solar panels. If there are water wells, we might help ourselves and others to have water. Even if there is no water, we will still be able to manage the water out of the wells. If this happens, we don’t need electricity. We just need solar panels.” Woman, 3rd PAR meeting*

During the third PAR group, the decision was made to mobilize resources to equip existing wells in the area with solar power. Focus group discussions with community members reflected the consensus support for the decisions made in the PAR groups and emphasized the value of installing solar panels as a solution:

*“We are told that you will construct a bore, but we agreed to install solar panels as there are too many wells but of no use.” Man, FGDs*

At this juncture in the PAR process, some men proposed places where water systems could be improved to the benefit of most people in their community:

*“Many people visit the Mosque; they wash their hands and ablution. So, if we have proper water systems at the Mosques many people will benefit from it.” Man, 3rd PAR meeting*

Following the third PAR meeting, additional meetings were conducted with community members and members of the local NGO with the purpose of identifying key wells with a strategic location and which would benefit a great proportion of the community if solarization was installed. A total of five wells located in different regions across the community were equipped with solar panels to enable water pumps to extract fresh water.

Following installation of the solar panels, PAR members, CHCs, and community members reflected on their impact on reducing COVID-19 transmission by providing them with access to clean water to comply with ablution and clean themselves more regularly. Benefits beyond the pandemic were also described:

*“It has dramatically helped us in this pandemic as the water was less and we could not fulfil the need for cleanliness. Due to this, there was a great chance of spreading this disease. Now due to the abundance of water, we fight this disease.” Woman, FGD*

*“There are wells, but water extraction with old techniques is complicated. Electricity is available only for 4 hours in 24 hours in this area. But due to solarization, the water supply becomes common, and whenever things become familiar, its use is also every day.” Man, FGD*

The solar panels were also perceived as a welcome asset of benefit to the whole community, and it was appreciated that these were installed where people needed them most. It was clearly acknowledged that the PAR groups had played a pivotal role in this:

*“With the help of the PAR members, it was discussed that there was no water for drinking, so they put solar panels in the Bazar of (Name of Village) which is an important and main place in the area.” CHC, FGD*

More freely available water also seemed to have wider community benefits regarding mutuality, improved social and domestic relations and general happiness:

*“They would say we cannot give you water as we struggle for it. Now, people won’t stop you from using water whenever you go. Due to panels, our houses and children remain clean. When our men would come to the homes before, we would ask them to bring water. They would say, you finished water which we got back. Our people, including men and women, are all happy.” Woman, 5th PAR meeting*

Despite such improvements however, the problem of water poverty was not solved for all members of the community, and that further installation of solar panels was needed:

*“It is still challenging to serve water as the panels are still insufficient to serve the requirements.” Man, FGD*

It was understood that the solar panels/wells required care and maintenance into the future and that regular use had to be arranged. It was further accepted that this was the collective responsibility of the whole community:

*“We will fix it collectively if it gets out of order, just as we nominated (Name of person in the community) as an elder. He will collect the fund by sending a person from house to house for fixing the panels.” Man, FGD*

### Resource mobilization: personal protective equipment

The second factor that PAR participants highlighted that would enable the community to adhere to COVID-19 public health advice was the lack of resources among the community to purchase the recommended personal protective equipment (PPE), e.g., face masks, soap, hand sanitizer.

A total of 193 PPE kits were distributed among families of children attending to the local school the by CHCs. The content of the PPE kits was chosen by members of the local NGO and researchers in Pakistan, following conversations of the community needs. Kits included the following items: sanitizer, face masks, tissues, toothpaste, toothbrush, nail cutter, combs, antiseptic soap, shampoo, washing soap, dust bin, ewer [large jug to carry water], loofah [local brush used for dusting], wiper [used to clean up any water or liquids on the ground] and a towel.

Prior to this provision it was highlighted that many people living in the community were unable to afford such products, especially at the beginning of the pandemic when demand meant prices were high. Proposed solutions included re-using or washing face masks and utilizing more affordable options such as making their own sanitizer or using mud and clay to sanitize their hands. Some of the financial barriers to uptake of PPE were eventually ameliorated by the free distribution, as decided upon by the PAR groups:

*“We would use one mask for a week, but the hygiene kit provided by (name of local NGO) benefitted us.” Boy, World Cafe*

*“A small bottle of Dettol costs us 120 Rs. That person told me that there is a formula which is using boiled water with salt and rose petal water, hydrogen, and a small bottle of spirit I diluted each one of these ingredients and made a sanitizer out of it at home and I distribute it among everyone. It was better than the actual sanitizers themselves. It was better in every sense, smell wise, and taste-wise.” Man, 3rd PAR meeting*

The PPE was well received by the community members. Some mentioned that elements of the kit were not usually bought and in some cases were even new to them given their cost or previous lack of awareness of their importance:

*“The cleanliness kit contained essential items, the importance of which was not known to the public. For example, they introduced toothpaste, brush, soap, and towels, which was unknown to this area’s people.” Man, FGDs*

The participants of the PAR meetings and FGDs believed that this provision helped in reducing COVID-19 infection risk, but also protected them from other diseases and health conditions. Appreciation for the value of these items extended to a commitment to carry on using them in the future and acknowledgement of the role of hygiene in enabling participation in community and cultural traditions:

*“Alhumdo LilLah [Arabic meaning 'Thanks to Allah’] provided us with masks and toothpaste, which eradicated many diseases. It’s not us, but our children who also follow their instructions. Wherever they go, they ask for masks and sanitize their hands. We would not go to the funerals before, but now we wear masks, sanitizers and everywhere. May Allah bless you.” Woman, 4th PAR meeting*

Women expressed their intention of requesting their husbands to continue to buy the products contained in the PPE kits:

*“If we ask our men, they will buy all the essentials included in the kit, whether masks, sanitizers, or soap. When we attended funerals, we would be astonished to see the people using sanitizers. Now we know its importance, and our men will bring it when we ask them to.” Woman, FGDs*

### Spaces where collaborative effort can bring to solutions

Community participants often looked beyond the pandemic to contemplate broader matters of health inequality and unmet needs. PAR members and FGDs participants expressed their concerns regarding other public health and social issues that needed to be solved within their community including the need for more medical resources, infrastructure, improved personal security and prevention of dengue and drug misuse.

Across the community, there was a clear consensus that the PAR meetings allowed a space where community members could discuss community issues and identify solutions to their problems. There was a collective appeal to continue with these meetings beyond completion of this specific project, such that a legacy would be ongoing inclusive of community involvement in decision making, especially regarding health and education:

*“We will be unfortunate if it is not extended. What will we do? There should be a school for girls till class 10. There should be a lady doctor in the hospital because many women have died. So, if this project does not extend, what will you do.” Woman, 5th PAR meeting*

It seems that the PAR process was congruent with other social and democratic characteristics of Pashtun culture, albeit one which includes the voice of women which is normally absent from traditional decision-making fora. It was noted how the valuable deliberations within the PAR context could be integrated with other community dialog and debate in traditional meeting places:

*“In Pashtun culture the people meet at night and talked about what happened during the day. As the PAR members consisted of people from different areas and they would discuss the issues in different communities, this brought a positive change in the attitude of people. They would praise those who would use masks and follow SOPs. The PAR members have a great influence on the people.” CHC, FGD*

Men and women participating in the focus group discussions were also keen to get involved in such extensions to community democracy:

*“We will make a group if there is a project or not, and we will discuss different issues in that group, and with the joint suggestion, we will work for the welfare of this area.” Man, FGDs*

This democratic appeal was inclusive of women’s voices:

*“We discussed all our problems very openly and without hesitation. We discuss everything with (Name of Research Assistant) like an elder sister even if you cannot fulfil our demands. We still discuss it with you, and we want these meetings to continue discussing our problems.” Woman, FGDs*

During these discussions, various ways of organizing inclusive involvement in community decision making were proposed and, in an area prone to natural disasters, the potential to contribute to community responses was recognized:

*We need to make a team of 8 to 10 members from (Village Names) who can deal with disasters. We should have such a force that can deal with an emergency. Each should prepare 6 to 8 people in their neighborhood to have a team of 50 people who can help us in emergencies …. There will be teamwork in a disaster like earthquakes, fire, or any endemic. In our next meeting, he will give the names of those five persons, and they will be the members of his team, and every member will be the leader of his team. This will provide us with great support.” Man, 5th PAR meeting*

### Agents of change

Participation in the PAR process brought various benefits for individual participants. These people began to perceive themselves as role models during the programmed, and acted as conduits of information exchange for their community, such as in the promotion of preventive behaviors:

*“We should talk to people and make them aware of it by telling them about the relevant SOP’s. So, with this people will become aware of it. Like telling people to apply sanitizers before leaving their homes, and masks.” Man, 3rd PAR meeting*

Subsequently, PAR members expressed that they had taken the knowledge obtained during the meetings and shared it with their families and social networks:

*“We gained awareness not only individually but collectively. Individually means I informed my own family about covid, and collectively as a community, we were aware and informed each other about the dangers of the corona [COVID-19].” Man, 4th PAR meeting*

Importantly, this included women acting as vectors for communicating key messages in the domestic sphere:

*“People would come to my house for stitching their clothes, and I would give them all information and tell them about everything. Also, we got many things from this jirga. We were not familiar with many things. We came to know about it and made use of it.” Woman, 4th PAR meeting*

Participants realized they could have an important role in helping their community and spoke of role modeling safe behavior and taking actions to promote public health messages and vaccination:

*“Wherever I go somewhere, I sanitize my hands and my friends. Most of my friends borrow sanitizer from me after shaking hands with others.” Man, 4th PAR meeting*

Participants grew in confidence such that the protective value of hygiene practices could be performed with marked symbolism, enhancing influence, and bringing widespread affirmation from peers:

*“We were playing cricket and one of the men from the audience shook his hand directly to me. I stopped the match and sanitized my hands and the whole community clapped for me.” CHC, FGDs*

CHCs believed that many people would listen to their advice on vaccination and the importance of following public health guidance to protect their families against COVID-19, and this contribution to community safety engendered substantial satisfaction with their work:

*“This project was full of enjoyment for us. We ourselves learned a lot of things when people followed our instruction it gave inner satisfaction due to our efforts people got vaccinated. Allah says saving a person is like saving the whole of humanity. This is a great achievement for us, and we think we have saved everybody. It is a proud moment for our team.” CHC, FGD*

### Empowerment of women

The CHCs believed that women in the community commonly do not have access to health information and that visiting women in their homes and providing them with such information gave them the necessary tools to reduce their risk of disease and encourage them to vaccinate:

*“As I told you that the women were illiterate, and we started from zero. But when they attended the PAR meetings, they said salaam instead of shaking hands and used masks and sanitizers.” A CHC, FGD*

This was confirmed by women participants who believed that the information learned during the PAR meetings and meetings with the CHC, gave them the necessary knowledge and resources to protect their families:

*“We became aware of many things which we were not familiar with before. We are housewives and busy all the time with household activities. But when we came here, we came to know the true meaning of cleanliness. We got much awareness from this meeting and are very thankful for your invitation and listening to our problems.” Woman, FGDs*

To some extent women felt that being part of the project allowed them to have their opinions valued at home:

*“We pray for you and are thankful for your project. We don’t want you to stop these meetings.. We run the houses as men are outside, and we look after our children. Before, our men would not listen to us, but due to participation in these meetings, they have realized that we have our say.” Woman, FGDs*

## Discussion

This community-based participatory action research carried out in a rural community in Pakistan during the COVID-19 pandemic, revealed several challenges facing such communities which limited their ability to follow public health guidance to limit the spread of infection. Nevertheless, engaging members of the community in the PAR process afforded opportunities to harness people’s agency and capacity to generate solutions for such problems. This supports evidence that participatory approaches lead to real impact in developing public engagement to promote COVID-19 emergency preparedness and risk communication among vulnerable groups (21). Crucially in our study, a positive hook for this engagement, and proving instrumental in actioning solutions, was the availability of resources within the project which could be mobilized under community control. The deliberative communicative practices of PAR, and consequential public health messages, proved to be congruent with local Pashtun culture and beliefs. There was widespread appreciation for the impact of this project, and a desire among participants and other community members for the PAR groups to carry on as a useful addition to community democracy. It should also be noted that our study did not operate in isolation to other public health measures being implemented at that time. The Pakistan government set up a national RCCE strategy that consisted of six interconnected streams: real-time policy statements; public service messages, ensuring two-way dialog; reaching the hard to reach; caring for frontline health workers; and rumor management (22). Although there was little evidence that our community was aware of the government’s RRCE activities, it is possible that the positive changes of the community toward the COVID-19 pandemic may be a combination of strategies that were taking place to reduce the spread of the pandemic.

Water, sanitation, and hygiene measures such as regular hand washing was central to the public health measures promoted by WHO to reduce the risk of transmission of infectious diseases, including COVID-19 (23–25). Early in our process it was revealed that the community had limited access to water and hygiene products, and that was one of the main barriers to following the WHO recommended behaviors (23). The limited access to WASH facilities among families living in rural deprived areas in Pakistan is well described (26, 27) and this predisposed such communities not only to infectious diseases, but also to adverse outcomes such as malnutrition, psychosocial stress, poor birth outcomes, lower cognitive function, and performance, among other health and financial outcomes (28). Findings from this study highlighted the vulnerability of rural communities with low access to WASH during the pandemic. This limited access to water among the community posed a serious public health concern even before the COVID-19 pandemic (28) and therefore, in order to achieve the UN’s 2030 agenda, it is crucial that policy makers and NGO’s work together with communities with low access to water to find sustainable solutions to increase access to WASH facilities.

At the beginning of our project many members of the community voiced skepticism toward the existence of the COVID-19 pandemic and safety of the vaccine. Previous health crises have highlighted that mistrust and rumor can undermine public confidence in the scientific evidence and can be a dangerous hindrance to response efforts. Our findings are in alignment with other recent studies (29, 30) that have shown that the COVID-19 pandemic was accompanied by an infodemic of false and misleading information (29) triggering confusion, public anxiety, and risk behaviors (31). We found that these initial feelings of skepticism changed through the course of the project, often attributed directly to the PAR process. That said, the established involvement of a trusted NGO in the area, with a development focus on health and education, was also supportive in consolidating trust in public health advice. This study supports existing evidence that suggests that partnering with the community and involving community leaders favors the uptake of pandemic control measures (12, 32–34).

Our study demonstrated that partnering with male and female community members in a democratically participatory process enabled the identification of practical solutions to issues that were perceived to be the most urgent to be addressed. Discussions with the wider community revealed that this approach was found to be a beneficial and trusted mechanism with which to make positive changes within the community. Key to the success of the participatory process was the equal involvement of women on decision making. The Global Gender Gap Report 2020 ranked Pakistan at 151 of 153 countries in regard to gender equality. Women face gender inequality at home, in education and within the workplace, with less than half of women being literate and only a quarter participating in the labour force (35). It is widely recognized that girls and women bore the brunt of the pandemic, which has exacerbated pre-existing gender inequalities (36). The equitability of our participatory approach was particularly important since the knowledge, experiences, and concerns of women in such communities are not usually explored or considered in decision making due to the cultural role of women in rural Pakistan and within a predominantly patriarchal Pashtun culture. A previous study in Northwest Pakistan evidenced that men and religious leaders are the key decision makers when it came to mobilization of resources and community matters including health, whereas women have very little decisional or influential powers (37). It was clear from our qualitative findings of PAR deliberations that women made valuable and well-received contributions to this process. Incorporating issues of women and gender that have been lacking in previous emergency responses and could lead to enhanced community resilience.

The opportunity for PAR participants to go beyond discursive reflection on issues facing the community and involve themselves in democratically deciding how resources could be mobilized was a notable aspect of this project. The findings strongly suggest that there was a helpful compatibility between the democratic principles and practices of PAR and certain characteristics of Pashtun culture and Islamic faith. For example, the cultural tradition of Pashtun jirga, an essentially deliberative system for adjudicating community decisions and mediating conflict, has congruency with intended participatory democracy of PAR. Such observations have also been made in previous community health projects in the region (23). In conjunction with community affinities for deliberative decision-making, key tenets of Islamic scripture are supportive of reliance on scientific-medical opinion as long as these are accepted as Allah’s will. In this regard, individual and collective agency to decide upon positive public health measures can transcend any tendency to ground a fatalistic acceptance of disease and illness in a simplistic faith in preordination of outcome. Arguably, this cultural congruence, alongside the undoubted effectiveness of the specific hygiene strategies, was influential in an expressed desire for this sort of community forum to persist beyond the completion of this project (22).

### Conclusions and further work

Whilst RCCE has been acknowledged as a key component in public health response to emergencies, the success of such strategies relies on strong partnerships and engagement among affiliated groups. It has been highlighted that more emphasis should be placed on strengthening local structures and communities to ensure their active participation in interrupting disease transmission (38). The participatory approach of our research was integrated with local culture and values and provided a mechanism which enabled the community to explore the challenges they faced in reducing the spread of infection and engaged them in finding sustainable solutions. The formation and continuation of self-managing community forums enable ongoing grass-roots community input into developing health and education initiatives.

The significance of the findings generated by this study is that they provide a greater understanding of the impact of participatory processes in emergency contexts and offer insights with the potential to improve the responses to other public health and climate change crises. Our work has fed into regional government and NGO RCCE activities, both during the pandemic and beyond. Members of our team have joined with UNICEF Pakistan to provide RCCE training to religious leaders, health care professionals and civil society organizations in Khyber Pakhtunkhwa, with particular focus on vaccine uptake and disaster preparedness in response to the recent floods in Pakistan.

## Data availability statement

The raw data supporting the conclusions of this article will be made available by the authors, without undue reservation.

## Ethics statement

Ethical approval for this study has been granted by the University of Central Lancashire (HEALTH 0105) and Khyber Medical University (DIR/KMU-EB/IC/00033). The patients/participants provided their written informed consent to participate in this study.

## Author contributions

VM, MM, SF, UM, MC-R, and MZ contributed to the design and development of the study. MZ provided overall management for the project. SF managed data collection. UM checked the accuracy of the transcripts. SA ran the PAR meetings and focus groups. MC-R and UM completed initial coding and analysis of the data, which were double checked by VM and MM. All authors contributed to reviewing and interpreting key themes. MC-R and VM wrote the manuscript. VM and MM revised the paper and finalized the manuscript. All authors have read and agreed to the published version of the manuscript.

## Funding

This research was funded by the UKRI GCRF/Newton Fund Agile Response call to address COVID-19. Grant Title: GCRF_NF225 - Improving community engagement with COVID-19 public health messages in hard-to-reach communities. Grant ref: EP/V04320X/1.

## Conflict of interest

The authors declare that the research was conducted in the absence of any commercial or financial relationships that could be construed as a potential conflict of interest.

## Publisher’s note

All claims expressed in this article are solely those of the authors and do not necessarily represent those of their affiliated organizations, or those of the publisher, the editors and the reviewers. Any product that may be evaluated in this article, or claim that may be made by its manufacturer, is not guaranteed or endorsed by the publisher.

## Abbreviations

CHC: Community health champions
FGDs: Focus group discussions
PAR: Participatory action research
RCCE: risk communication and community engagement
WASH: water, sanitation, and hygiene
