# Supplementary material for: Participatory action research to co-design a culturally appropriate COVID-19 risk communication and community engagement strategy in rural Pakistan
Source: Front Public Health. 2023 Apr 24;11:1160964. doi: 10.3389/fpubh.2023.1160964 (PMC10166109; doi:10.3389/fpubh.2023.1160964)
Supplement: Supplementary file 1 [file Table_1.docx]

Supplementary Material

Participatory action research to co-design a culturally appropriate COVID-19 risk communication and community engagement strategy in rural Pakistan

Victoria Hall Moran^1^, Marena Ceballos-Rasgado^1^, Sadia Fatima^2^*, Usman Mahboob^3^, Salman Ahmad^4^, Michael Mckeown^5^, Mukhtiar Zaman^6^

^1^Centre for Global Development, University of Central Lancashire, Preston, UK

^2^ Institute of Basic Medical Sciences, Khyber Medical University, Peshawar, Pakistan

^3^Institute of Health Professions Education and Research, Khyber Medical University, Peshawar, Pakistan

^4^ Department of Sociology, Abdul Wali Khan University Mardan, Pakistan

^5^School of Nursing, University of Central Lancashire, Preston, UK

^6^Department of Pulmonology, Rehman Medical Institute, Peshawar 25000, Pakistan

*** Correspondence:**Corresponding Author
sadiafatima@kmu.edu.pk

# Topic guide Focus Groups with community members

**[Facilitator to give a brief introduction to the project]**

1. Were you aware that this work has been done?
2. How did you find out about the project?
3. Do you feel involved in this project? If so, how?
4. **Hygiene Kits**
5. Have you used the hygiene kits?
   1. If yes, how did you use them within your household/families?
   2. If no, why not?
   3. Do you think that they affected how you are coping with the pandemic? If so, in what way? If not, why not?
6. Are you still taking precautions to minimise your/your family’s risk of catching the virus? If yes, what? If no, why not?
7. What are your future plans to use the hygiene kit once the project is finished?
8. **Solar Panels Utilization**
9. Have you used the solar panels? If yes, tell me about your experiences of using them? If no, why not?
10. Have they influenced how you are coping with the pandemic? If so, in what way? If not, why not?
11. In your opinion, who has responsibility for maintenance of the solar panels? Why do you think so? What do you think needs to be done to maintain them?
12. **PAR members responsibility**
13. Did you have any involvement in the project?
    1. If yes, tell me how you were involved.
    2. If no, why do you think this is? Would you have liked to me more involved?
14. Have you heard about the PAR meetings? If yes, tell me more about what you know about them and how you know about the group and its activities (eg do you know one of the members? In what ways might people like yourself get to hear about this sort of thing & how might you feed in your views/ideas?).
15. Do you think the members of the PAR groups share your views? If not, why not?
16. Do you think the PAR groups did a good job of identifying community needs?
17. Do you think is there a need of this group to carry on their activities?
    1. If yes, what do you think what would be required for the group to carry on such activities?
    2. If no, why not?
18. Do you think that this project has helped you to be more prepared for any future emergences like Dengue or another pandemic for example? If yes, how. If no, why not?

# Focus Groups with community health champions

1. Can you tell us about your job as a community health champion? Give us an example of the kind of things you did.
2. What did you enjoy most about being a community health champion (overall)?
3. Did you learn anything new? If so, what?
4. What was the most difficult part of your role as a community health champion (overall)?
5. Were you able to do something to overcome these difficulties?
6. Has your understanding of COVID-19 been the same before and after this project? If yes why and how, if not why?
7. Has this project influenced you personally? Why do you think so?
8. Have you changed your behaviour to avoid infection as a result of being part of this project? If yes why and how, if not why?
9. Have you noticed any changes within the community since this project started? Why do you think so?
   1. Do you believe that the community understanding of COVID-19 changed their behaviours among their neighbours? (Hygiene, vaccination uptake, etc.)
   2. Have you worked in anyway with the PAR participants, and if so how?
   3. Are the people participating in the PAR groups have become change agents? How?
   4. Do you communicate with the Jirga/ Abaseen foundation, community elders, hujra
10. Have you received any feedback from the participants about the project? What is that?

# World Café with Children Key questions/points:

1. What children know about COVID-19. Where did they learn it from?
2. Children’s perception of their vulnerability towards coronavirus.
3. Explore children’s understanding of the severity or seriousness of coronavirus.
4. What actions at the individual level are children taking to avoid infection?
5. What actions at the family level are being taken to avoid infection?
6. Are they aware of the actions being taken by the community to avoid infection?
7. Have they noticed any change in the water sources? What effect has this had in their day-to-day life? As children, what can they do to care for the new sources of water?
8. Did they use the kits provided?
9. Have they used the PPE provided during the project? Have they done anything different?

**
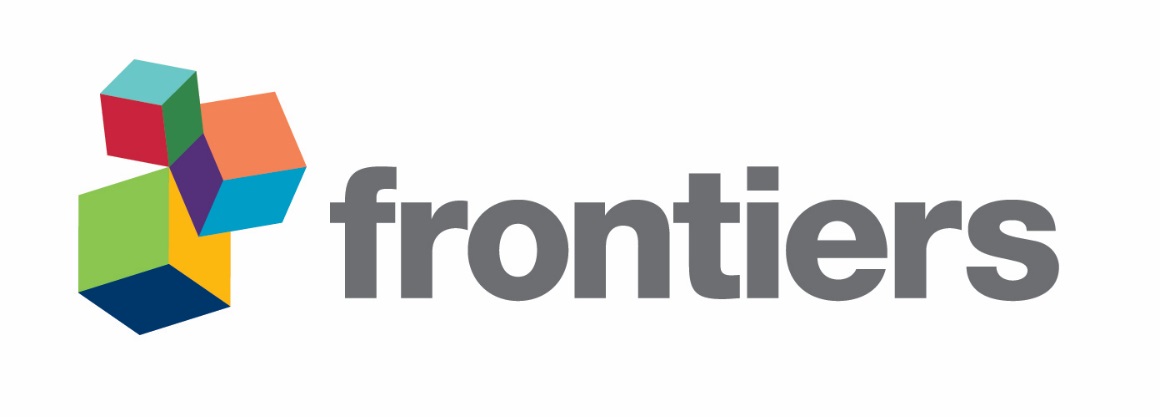
**
